# A Case of Acquired Factor V Inhibitor During Bullous Pemphigoid Treatment

**DOI:** 10.7759/cureus.69090

**Published:** 2024-09-10

**Authors:** Kaho Ou, Haruka Arakawa, Yui Togashi, Hiroyuki Fujita, Setsuko Matsukura

**Affiliations:** 1 Department of Dermatology, Saiseikai Yokohamashi Nanbu Hospital, Yokohama, JPN; 2 Department of Hematology, Saiseikai Yokohamashi Nanbu Hospital, Yokohama, JPN

**Keywords:** acquired factor v inhibitor, anti-factor v antibodies, autoimmune disorder, bullous pemphigoid, coagulation disorder

## Abstract

Acquired factor V inhibitor (AFVI) is a rare coagulation disorder caused by the production of anti-factor V antibodies in response to infection, surgery, malignancy, autoimmune disease, antibiotics, or other drugs. Its clinical manifestations vary from asymptomatic to severe; hence, optimal treatment is difficult. Bullous pemphigoid (BP) is an autoimmune disorder caused by autoantibodies against dermal-epidermal junction structural proteins. We describe a patient with BP and AFVI, successfully treated with prednisolone. A 78-year-old Japanese man presented with tense hemorrhagic blisters on his trunk and extremities. Owing to his urinary tract infection and advanced age, oral prednisolone was initiated at 20 mg (0.3 mg/kg/day) for BP. Three weeks after treatment, upper gastrointestinal bleeding, hemorrhagic shock, aspiration pneumonia, and hematuria occurred. An elevated anti-BP180 antibody titer (2050 U/mL) indicated BP, and a prolonged activated partial thromboplastin time (aPTT, >180 seconds) indicated a coagulation disorder; the international normalized ratio was too prolonged to be calculated. Based on low factor V activity (<1%) and an inhibitor pattern in an aPTT cross-mixing test, we diagnosed possible AFVI. Despite BP and AFVI stabilization, prednisolone administration (18 mg/day), and normal aPTT, the patient died of septic shock due to cholangitis. In conclusion, clotting-related tests, including factor V tests, should be performed if coagulation disorders persist during the treatment of autoimmune diseases such as BP. There is a hypothesis that immunoglobulin G4 is associated with AFVI and BP and that there is a homologous sequence between factors Ⅷ and V and the BP180 protein. This may explain the immediate resolution of the disease after prednisolone administration.

## Introduction

Bullous pemphigoid (BP) is an autoimmune disorder caused by autoantibodies (immunoglobulin G, IgG) against structural proteins of the dermal-epidermal junction. Tense blisters and erosions on the skin or mucous membranes close to the skin surface clinically characterize BP [1]. Generally, mucous membranes are only rarely affected by BP but are usually affected by mucous membrane pemphigoid.

Acquired factor V inhibitor (AFVI) due to inhibitor production is a rare coagulation disorder. Approximately 200 cases in which selecting the optimal treatment was difficult have been recorded globally, with varying clinical manifestations. These ranged from asymptomatic to severe hemorrhagic diathesis [2,3]. A quarter of cases are asymptomatic [4]. Factor V has two properties: procoagulant and anticoagulant. Most AFVI cases present with hemorrhagic symptoms, but if procoagulation and anticoagulation are balanced, the disease is asymptomatic. If anticoagulation is strongly suppressed, then thrombosis might occur [3]. The most common sites of bleeding (and subsequent hematoma formation) are the gastrointestinal tract and urinary tract, followed by the skin, upper respiratory tract, and intracranial and pulmonary sites. Reported treatments include corticosteroids, cyclophosphamide, azathioprine, rituximab, intravenous immunoglobulin, and plasma exchange [3].

Herein, we describe a patient with BP and possible AFVI, successfully treated with prednisolone. After 15 weeks, he passed away from septic shock due to cholangitis.

## Case presentation

**Figure 1:**
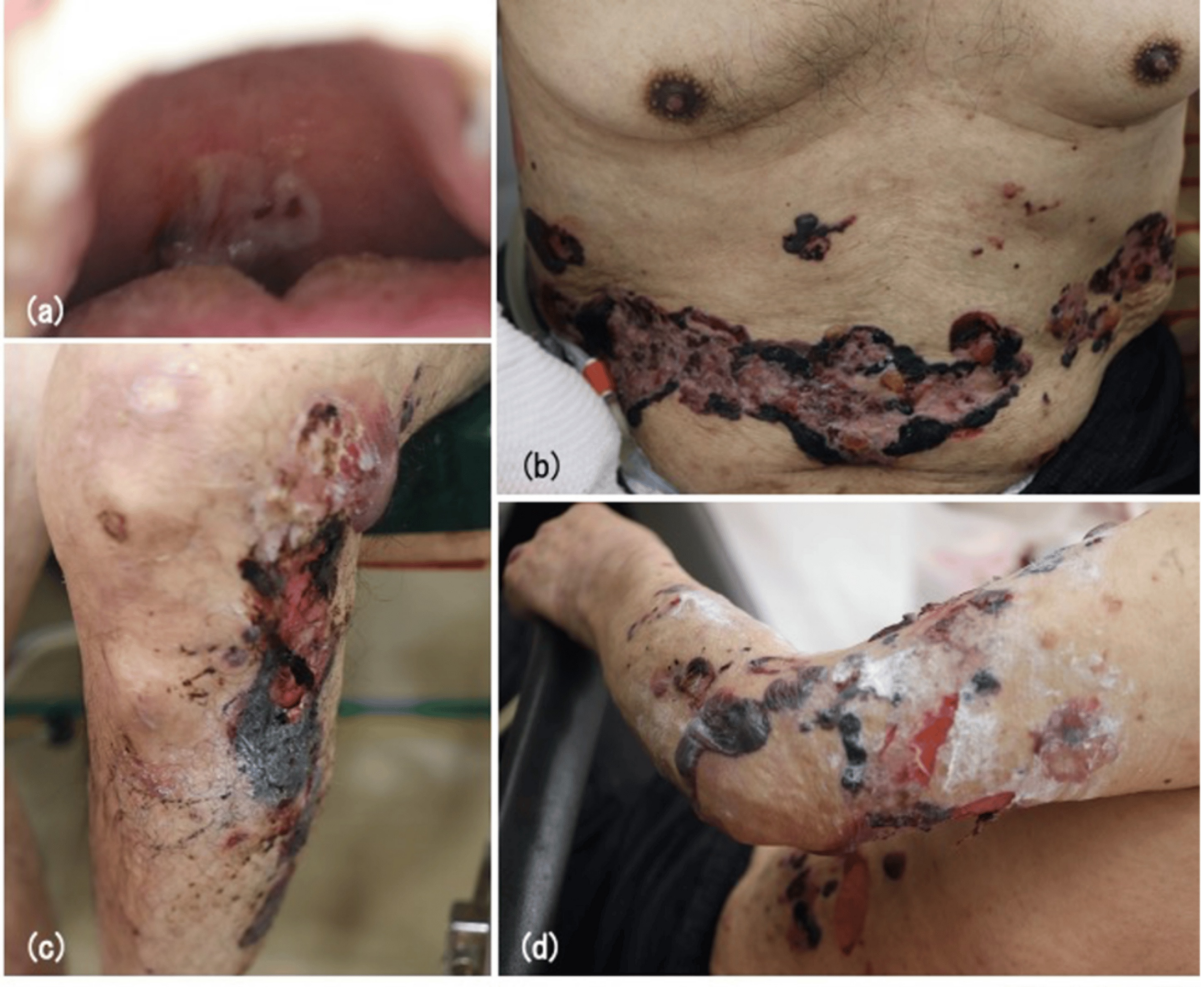
Multiple tense hemorrhagic blisters and erosions are seen on the patient's hard palate (a), abdomen (b), leg (c), and arm (d).

A 78-year-old Japanese man presented with multiple tense hemorrhagic blisters on his trunk and extremities. He had a history of cerebral infarction and received edoxaban tosylate hydrate (60 mg/day), which was initially thought to be the cause of hemorrhagic blisters. When the blisters had appeared on his trunk three months prior, dimethyl isopropylazulene, zinc ointment, and oral antihistamines were prescribed in another hospital. However, the blisters were intractable and spread throughout the body. Physical examination revealed multiple tense hemorrhagic blisters and erosions in the oral cavity, trunk, and extremities (Figure 1).

**Figure 2:**
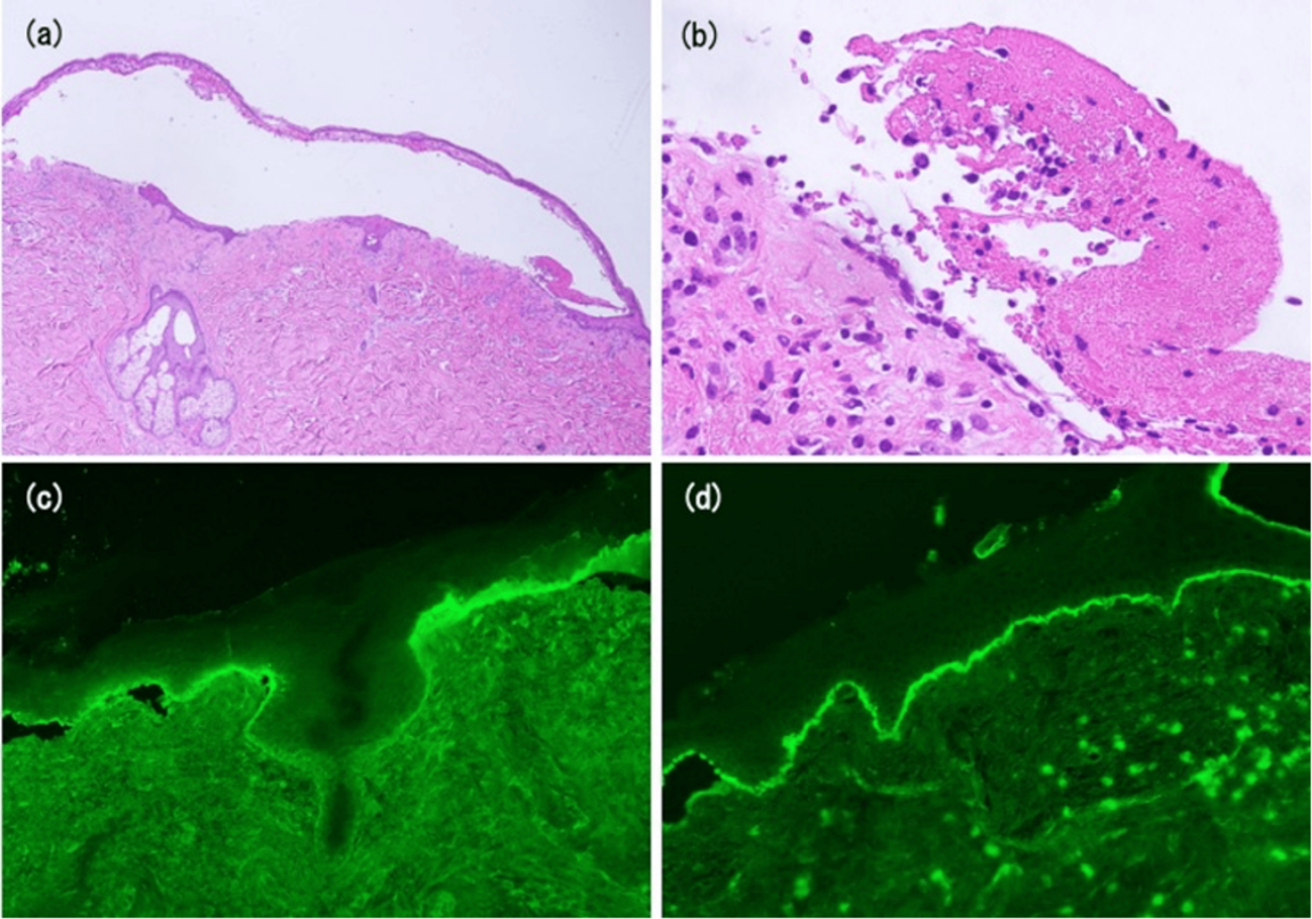
Histopathology of a biopsy from the abdominal blisters of the patient. Subepidermal blisters with eosinophil and lymphocyte infiltration (a, b): hematoxylin & eosin, ×40 (a) and ×400 (b). Direct immunofluorescence analysis reveals a linear deposition of immunoglobulin G in the basement membrane zone (c) and complement C3 (d).

The Bullous Pemphigoid Disease Area Index score (BPDAI) of the skin, erosions/blisters was 43; urticaria/erythema was 11; and mucosal, erosions/blisters was 2. Skin biopsy showed subepidermal blisters with eosinophil and lymphocyte infiltration (Figures 2a, 2b).

Direct immunofluorescence revealed linear IgG and C3 basement membrane deposits (Figures 2c, 2d). On chemiluminescent enzyme immunoassay, serum anti-BP180 antibody was 2050 U/mL; thus, BP was diagnosed. The prothrombin time international normalized ratio (PT-INR) was 1.95 (normal range: 0.9-1.15), and the activated partial thromboplastin time (aPTT) was 35 seconds (normal range: 24-32 seconds) on admission to our hospital. The patient also had a urinary tract infection; therefore, the corticosteroid dose was started at a lower dose; oral prednisolone was initiated at 20 mg (0.3 mg/kg/day). Subsequently, the skin lesions stabilized one week into the treatment course; the BPDAI of the skin, erosions/blisters, was 30; urticaria/erythema was 0; and mucosal, erosions/blisters was 0.

**Figure 3:**
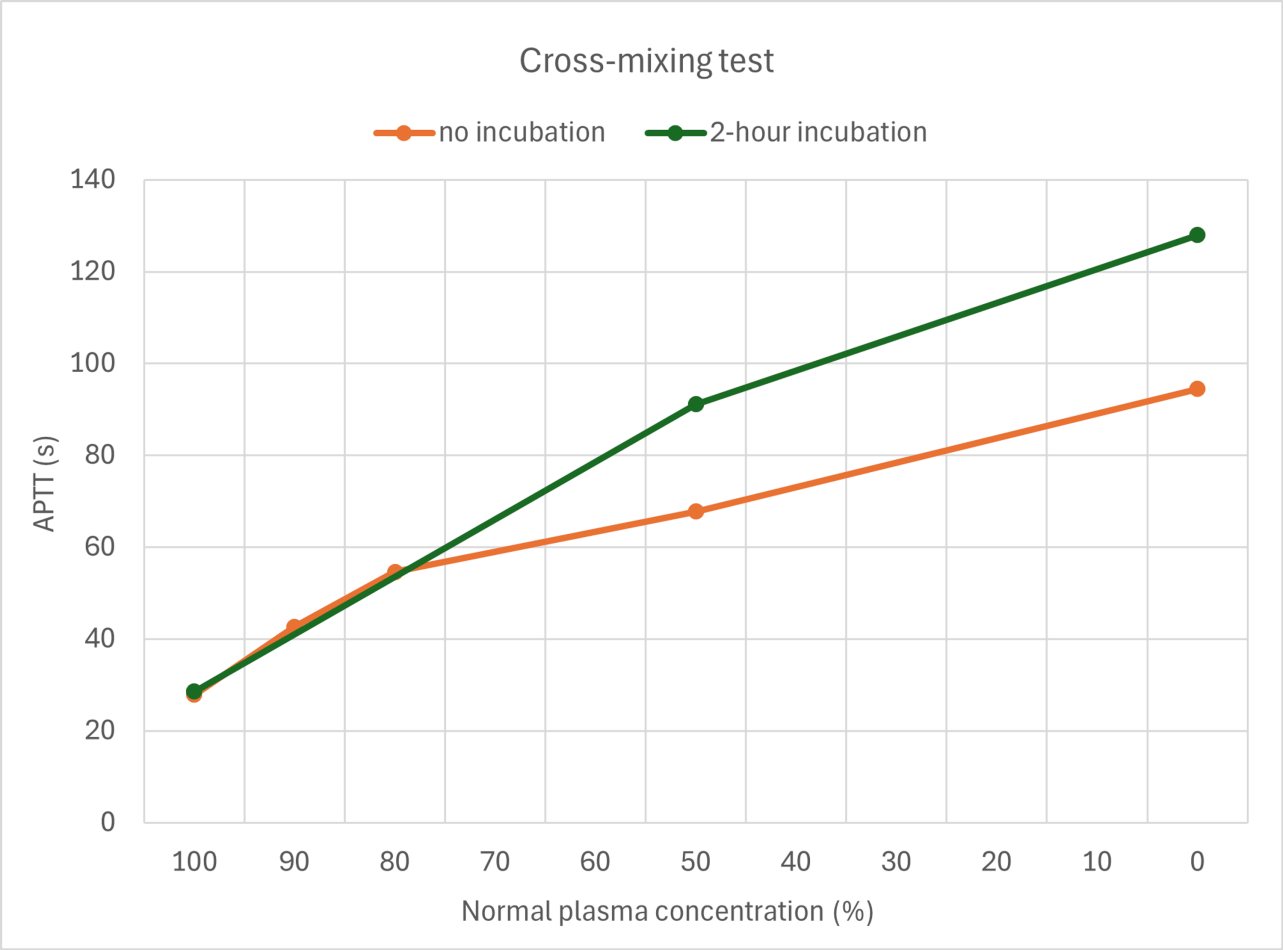
First aPTT cross-mixing test. The aPTT cross-mixing test of the immediate reaction (no incubation) and the reaction after two hours (2-hour incubation) showed an upward convex graph pattern, suggesting the presence of inhibitors. aPTT: activated partial thromboplastin time.

However, at three weeks, upper gastrointestinal bleeding, hemorrhagic shock, aspiration pneumonia, and hematuria occurred. The PT-INR was too prolonged to be calculated, and the aPTT was >180 seconds, indicating a coagulation dysfunction. Based on low factor V activity (<1%) and an inhibitor pattern in an aPTT cross-mixing test (Figure 3), we diagnosed possible AFVI.

Four units of red blood cell concentrate and four units of fresh frozen plasma were administered. Subsequently, the patient developed a catheter-related Candida bloodstream infection and difficulty taking oral medications. Therefore, the treatment was switched to 30 mg/day of intravenous prednisolone sodium succinate. At six weeks, serum anti-BP180 antibody had decreased to 153 U/mL, without new blisters. At seven weeks, BPDAI of the skin, erosions/blisters was 5; urticaria/erythema was 0; and mucosa, erosions/blisters was 0. The PT-INR and aPTT improved to 3.72 and 67 seconds, respectively, but remained prolonged. Thus, the oral prednisolone dose was switched to 25 mg/day.

**Figure 4:**
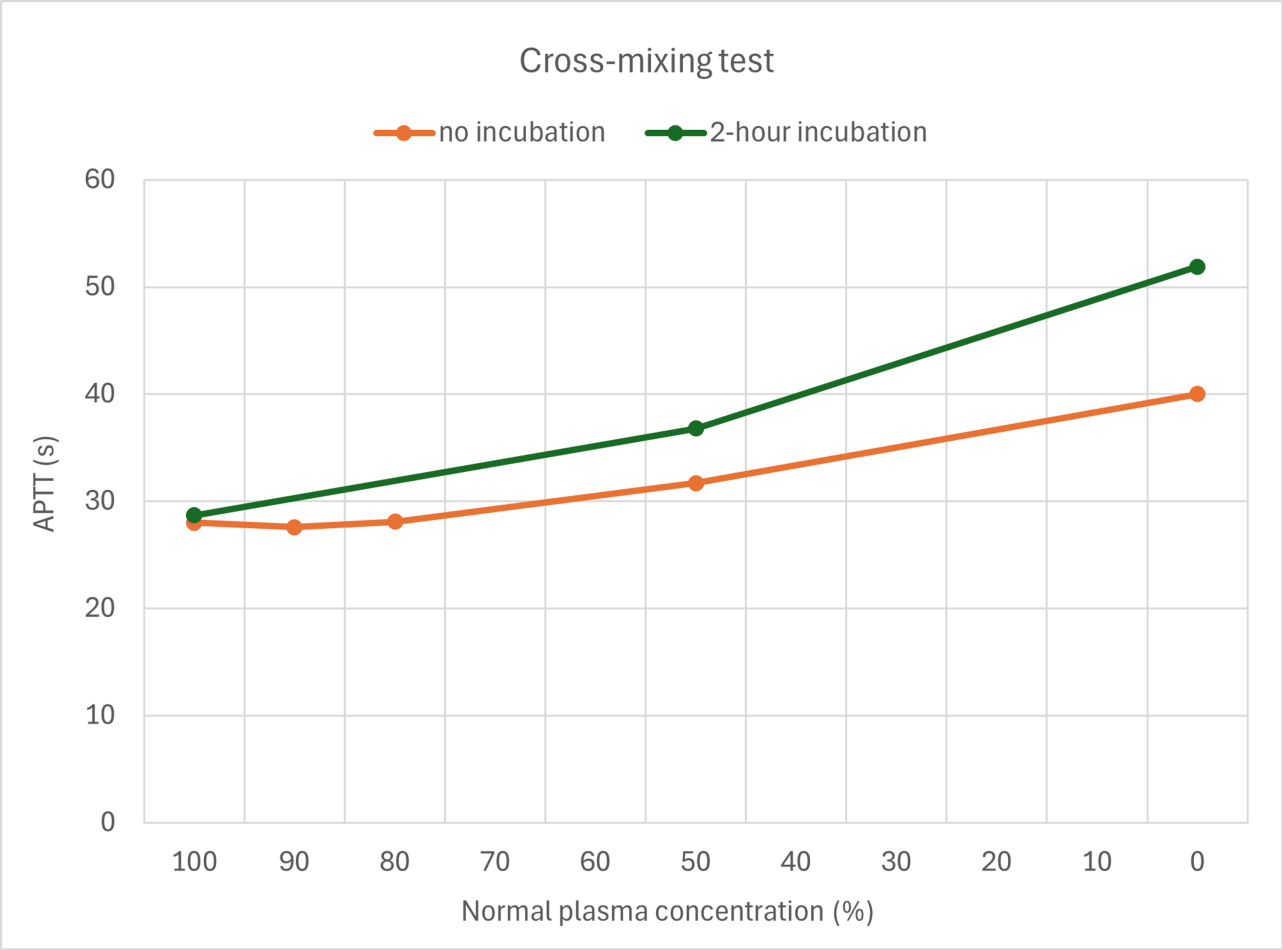
Second aPTT cross-mixing test. The cross-mixing curves transitioned from the initial upward convex inhibitor pattern to a less pronounced convex pattern, indicating a weakening inhibitor effect and improving coagulation status. aPTT: activated partial thromboplastin time.

At eight weeks, a repeat cross-mixing test confirmed improved coagulation; the cross-mixing curves transitioned from the initial upward convex inhibitor pattern to a less pronounced convex pattern, indicating a weakening inhibitor effect and improving coagulation status (Figure 4).

The patient's general condition improved, and he was discharged. However, at 15 weeks, he was admitted to the Department of Gastroenterology owing to cholangitis. Despite disease stabilization, post-discharge prednisolone administration (18 mg/day), and normal aPTT, he died of septic shock due to cholangitis.

## Discussion

**Figure 5:**
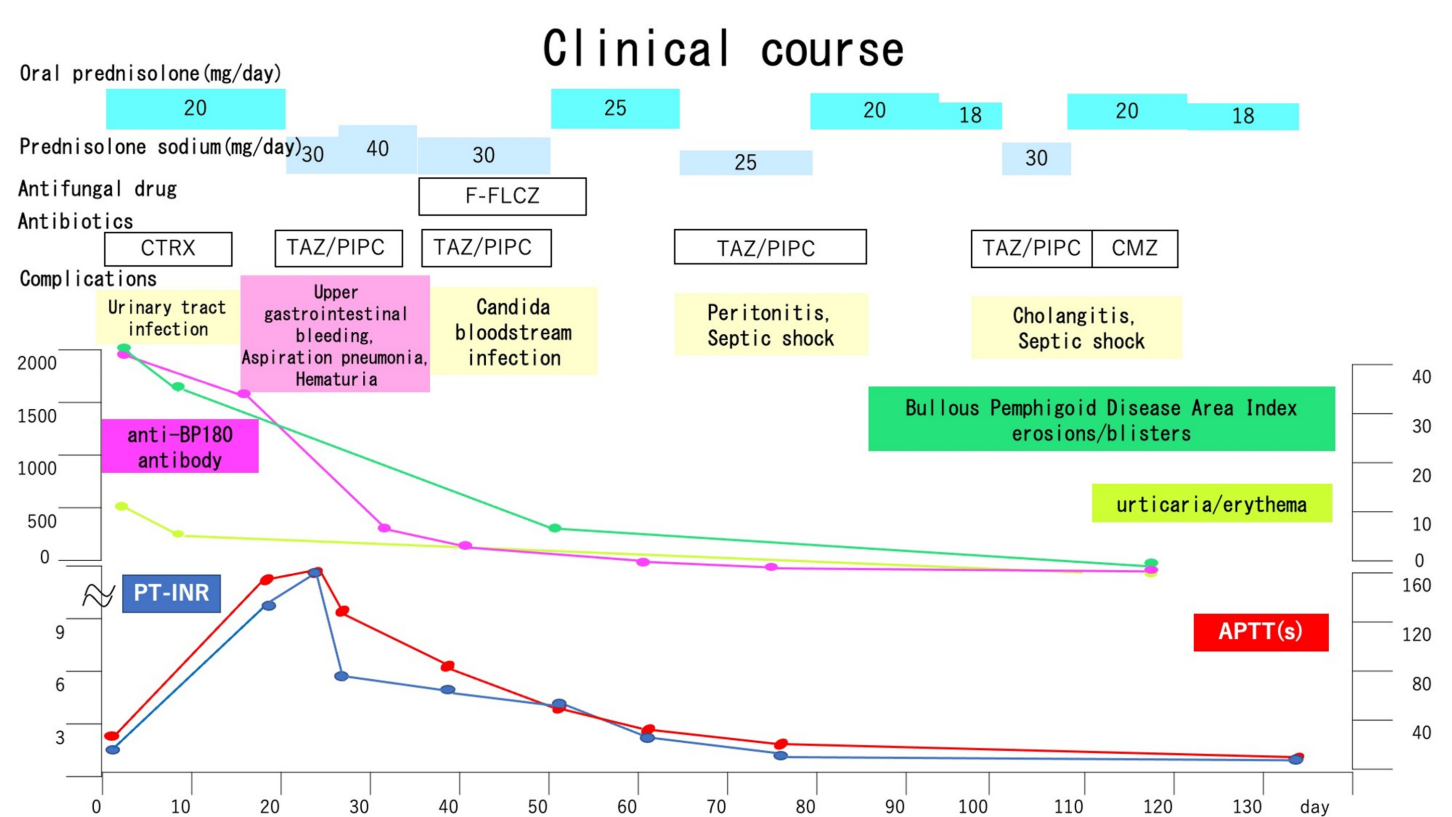
Clinical course of this patient at admission. Six weeks post-treatment, the serum anti-BP180 antibody concentration had decreased to 153 U/mL. CTRX: ceftriaxone; TAZ/PIPC: tazobactam/periperacillin; F-FLCZ: fosfluconazole; CMZ: cefmetazole; PT-INR: prothrombin time international normalized ratio; aPTT: activated partial thromboplastin time.

AFVI is caused by infections, antibiotics and other drugs, surgery, malignancy, and autoimmune diseases [3]. The patient in our case had BP as an autoimmune disease and several infectious diseases, including urinary tract infections and aspiration pneumonia. During the clinical course, several drugs, such as antibiotics, including ceftriaxone and tazobactam/piperacillin, were also suspected to be triggers of AFVI (Figure 5).

The following hypotheses are possible regarding the mechanism by which AFVI and BP coexist. IgG, specifically monoclonal IgG4, with inhibitory activity against human coagulation factor V, was isolated from the serum of a patient with a fatal bleeding diathesis [5]. BP is caused by IgG against structural proteins of the dermal-epidermal junction, and the IgG subclasses are IgG1, IgG2, and IgG4 [6]. Not only IgG4 but also IgG1 may be related to the pathology. Thus, IgG is likely involved in both AFVI and BP. Furthermore, it is possible that epitope spreading occurred for IgG autoantibodies, leading to the concomitant occurrence of AFVI and BP.

In cases reported in the past, some authors appreciated that there is a sequence homology between factor VIII and BP180 proteins [7]. However, it has not been proven which part of the sequence homology exists. Moreover, it is said that the serum anti-factor Ⅷ antibody may interact with the central collagen-like part of the BP180 protein [7]. Furthermore, it has been reported in a patient with BP that the factor Ⅷ inhibitor was an IgG (IgG4 predominant) directed against the factor Ⅷ: C2 A2 domain [8]. Considering the homologous sequence (A1-A2-B-A3-C1-C2) in factors VIII and V [9], the anti-BP180 antibody may also have cross-reactivity with AFVI in our case. This hypothesis could explain why the disease activity of both BP and AFVI resolved almost simultaneously after the administration of prednisolone. The combination of acquired hemophilia A (AHA) and BP is clearly more frequently reported than the combination of AFVI and BP [10,11]. It is not yet clear why AFVI is a rarer complication in BP than in AHA, but it may be due to differences in the prevalence of AFVI and AHA.

## Conclusions

In our study, we experienced a case of possible AFVI during the treatment of BP, in which both symptoms were alleviated by treatment with prednisolone. Consistent with the improvement in BP symptoms, AFVI symptoms also improved, suggesting a link between BP and AFVI based on the presence of IgG4 and possible sequence homology between BP180 protein and factor V. In conclusion, clotting-related tests, including factor V tests, should be performed if coagulation disorders persist during the treatment of autoimmune diseases such as BP or infectious diseases. Although it is well known that acquired factor VIII inhibitors can occur during BP treatment, it is necessary to consider both acquired factor VIII inhibitors and AFVI.
